# Transposon Tn7 Preferentially Inserts into GAA•TTC Triplet Repeats under Conditions Conducive to Y•R•Y Triplex Formation

**DOI:** 10.1371/journal.pone.0011121

**Published:** 2010-06-15

**Authors:** Miriam Mancuso, Mimi C. Sammarco, Ed Grabczyk

**Affiliations:** 1 Department of Genetics, Louisiana State University Health Sciences Center, New Orleans, Louisiana, United States of America; 2 Department of Medicine, Louisiana State University Health Sciences Center, New Orleans, Louisiana, United States of America; National Institute on Aging, United States of America

## Abstract

**Background:**

Expansion of an unstable GAA•TTC repeat in the first intron of the FXN gene causes Friedreich ataxia by reducing frataxin expression. Structure formation by the repeat has been implicated in both frataxin repression and GAA•TTC instability. The GAA•TTC sequence is capable of adopting multiple non-B DNA structures including Y•R•Y and R•R•Y triplexes. Lower pH promotes the formation of Y•R•Y triplexes by GAA•TTC. Here we used the bacterial transposon Tn7 as an *in vitro* tool to probe whether GAA•TTC repeats can attract a well-characterized recombinase.

**Methodology/Principal Findings:**

Tn7 showed a pH-dependent preference for insertion into uninterrupted regions of a Friedreich ataxia patient-derived repeat, inserting 48, 39 and 14 percent of the time at pH 7, pH 8 and pH 9, respectively. Moreover, Tn7 also showed orientation and region specific insertion within the repeat at pH 7 and pH 8, but not at pH 9. In contrast, transposon Tn5 showed no strong preference for or against the repeat during *in vitro* transposition at any pH tested. Y•R•Y triplex formation was reduced in predictable ways by transposon interruption of the GAA•TTC repeat. However, transposon interruptions in the GAA•TTC repeats did not increase the *in vitro* transcription efficiency of the templates.

**Conclusions/Significance:**

We have demonstrated that transposon Tn7 will recognize structures that form spontaneously in GAA•TTC repeats and insert in a specific orientation within the repeat. The conditions used for *in vitro* transposition span the physiologically relevant range suggesting that long GAA•TTC repeats can form triplex structures *in vivo*, attracting enzymes involved in DNA repair, recombination and chromatin modification.

## Introduction

Friedreich ataxia (FRDA) is caused by an expansion of GAA•TTC repeats in the first intron of the frataxin (*FXN*) gene [1]. The normal repeat size in the FXN gene is 6 to 30 repeats. Most affected FRDA patients are homozygous for large expansions, usually greater than 600 repeats [1], [2]. Friedreich ataxia patients show a marked decrease in both frataxin mRNA and protein levels [1]. Frataxin reduction and disease severity and progression correlate with the size of the smaller expanded allele [3], [4]. The correlation is not perfect, in part, because the repeats display continued somatic instability. Whether the repeat expands or contracts is tissue-specific. GAA•TTC repeats tend towards contraction in most tissues examined [5]. However, disease relevant tissues of FRDA patients show a bias towards age-dependent expansion [6]. In a tissue culture model we have demonstrated rapid, continuous incremental expansion by GAA•TTC repeats in human cells [7].

Structures formed by GAA•TTC sequences have been implicated in both the repeat expansion, and subsequent *FXN* gene repression. Expanded GAA•TTC tracts may reduce frataxin mRNA by inhibiting transcription elongation directly [8]–[12], or indirectly by promoting heterochromatin formation [13], [14]. The asymmetric purine•pyrimidine (R•Y) nature of the GAA•TTC repeat makes it prone to secondary structure formation. The length of the GAA•TTC repeat, the local pH, available counter ions, as well as local superhelical density all play a role in the type of DNA secondary structure formed [15]–[17]. That reduced pH encourages the formation of triple stranded structures containing one polypurine and two polypyrimidine strands (Y•R•Y) by R•Y repeats has been extensively documented [15], [16]. In addition, owing to its low C•G•C content, Y•R•Y triplex formation by pure GAA•TTC repeats has been demonstrated at neutral pH [18], [19]. Furthermore, in the native A-rich genomic context, even short repeats such as (GAA•TTC)_9_ sequences, can readily form a Y•R•Y triplex [19]. To form intra-molecular triplexes an R•Y sequence also requires mirror symmetry [15], [16], [20], so interruptions in a repeat can constrain intra-molecular structure formation.

Interruptions in an unstable repeat sequence have a stabilizing effect on the repeats. We have demonstrated that even a single point mutation in a long GAA•TTC repeat will significantly reduce the rate of expansion in human cells [7]. Interruptions may generally serve to stabilize trinucleotide repeats by anchoring the two strands of the DNA duplex in proper alignment, preventing slippage [21]. Alternatively, interruptions may stabilize trinucleotide repeats by breaking up the repeat stretch into smaller functional lengths, thereby limiting the formation of DNA secondary structures. Indeed, a fully interrupted FXN repeat allele (GAAGGA instead of GAA) did not form secondary structures and did not inhibit gene expression [22].

We are interested in probing the role DNA structures may have in attracting enzymes involved in DNA repair, recombination and chromatin modification, as some of these may be involved in expansion of the repeat or transcription repression mediated by the repeat. Here we use the bacterial transposon Tn7 as an *in vitro* tool to probe whether GAA•TTC repeats can attract a well-characterized recombinase. Tn7 encodes five transposition genes (TnsABCDE) that in combination define two separate transposition pathways [23]. In the first pathway, TnsABC (the core recombinase) interacts with TnsD bound to a specific site, *attTn7*, to promote integration adjacent to the *glmS* gene providing a safe-haven for the transposon [24]. A second pathway uses TnsABC + TnsE to promote transposition to sites unrelated to *attTn7*. Recently, TnsE has been shown to interact with the β-clamp subunit of the bacterial DNA replication complex to direct Tn7 to insert into replicating DNA [25]. In the absence of TnsD and TnsE, transposition mediated by a hyperactive TnsABC* occurs into many different target sites with low selectivity [26]. However, this hyperactive TnsABC has also been shown to preferentially insert Tn7 adjacent to triplex-forming oligomers psoralen cross-linked to plasmid DNA targets [27], [28] and adjacent to an intramolecular Y•R•Y triplex [27]. This apparent targeting of structure led us to use Tn7 as a model system for DNA transactions triggered by structures intrinsic to GAA•TTC repeats.

Here we show that transposon Tn7 preferentially inserts into an FRDA patient-derived GAA•TTC repeat tract in target plasmids. Tn7 insertion was pH dependent and occurred in a region-specific and orientation-specific manner within the repeat. Tn7 did not preferentially insert into interrupted areas of the repeat tract predicted to be less likely to form structures. On the other hand, transposon Tn5 showed no preference for any part of the repeat tract and inserted at random in target plasmids at all pH conditions used. Plasmids containing a transposon interrupted GAA•TTC repeat showed a decrease in Y•R•Y triplex formation potential. However, reduced ability to form Y•R•Y triplex structures did not correlate with RNA transcript levels obtained during *in vitro* transcription.

## Results

**Figure 1:**
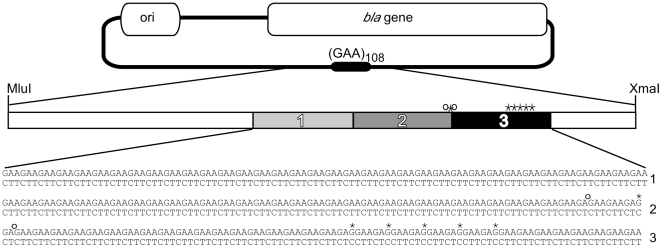
Experimental Target. Plasmid targets for *in vitro* transposition reactions contained 108 GAA•TTC repeats. Hollow boxes indicate essential regions (ori, *bla*) of the plasmid excluded from recoverable transposition events. The expected frequency of insertion into the (GAA•TTC)_108_ region based on its contribution to the size of the plasmid (minus the essential regions) is 9%. The repeat is located between restriction sites MluI and XmaI. The repeat is divided into thirds, and point deletions and substitutions in the GAA repeat are indicated by open circles and asterisks, respectively. The sequence of this repeat, divided into thirds, is shown at the bottom of the figure. The complete sequence of the insert is available online (GU722204).

In order to investigate the means and consequences of targeting GAA•TTC repeats for interruption, we chose to use a (GAA•TTC)_108_ allele already containing several point interruptions (Figure 1). This strategy provided several benefits. First, the interrupted (GAA•TTC)_108_ allele cloned into pBAD18 remained stable through repeated transfection and growth cycles in bacteria, in contrast to an uninterrupted (GAA•TTC)_88_ repeat in pBAD18 that we had worked with previously [12]. Second, the point interruptions provided landmarks for more easily determining transposon location within the repeat. Finally, the interruptions were largely clustered within the distal third of the repeat (Figure 1), enabling us to determine if these interruptions affected transposition within the repeat.

### Tn7 preferentially inserts into uninterrupted GAA•TTC repeats at lower pH

**Figure 2:**
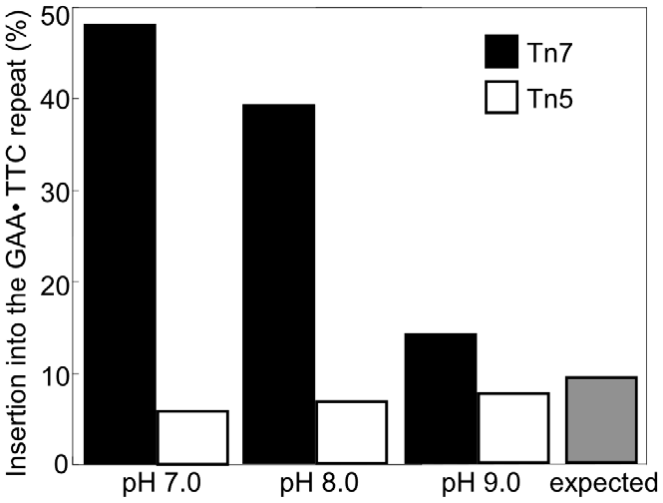
Preferential insertion into the (GAA•TTC)_108_ repeat by Tn7 is pH dependent. Bars indicate transposon insertion into the (GAA•TTC)_108_ repeat as a percentage of total insertions in the target plasmid at several pH values. Transposon Tn7 (black bars) shows a pH dependent bias for insertion into the repeat, inserting 48, 39 and 14 percent of the time at pH 7, pH 8 and pH 9, respectively. In contrast, transposon Tn5 is not attracted to the GAA•TTC repeat, inserting 6, 7 and 8 percent of the time at pH 7, pH 8 and pH 9, respectively. Insertion location was determined by a combination of restriction digest mapping and DNA sequencing. The expected frequency based on target size is 9% (gray bar). For pH 7 and pH 9 n = 96, for pH 8 n = 120.

We used the Genome Priming System (GPS-1) by New England Biolabs to achieve Tn7 transposition into target plasmids. The system uses Tn7 transposition proteins TnsA, TnsB and a hyperactive TnsC^A225V^ protein to achieve efficient *in vitro* transposition [26]. The transposon carried a selectable marker, so that plasmids containing Tn7 integrants could be recovered from transfected bacteria. The transposon donor plasmids we used had an R6K-γ origin of replication requiring specific host factors for replication [29], so that co-transformants do not complicate analysis. An initial restriction digest of miniprep DNA prepared from doubly selected transformed target plasmids was used to rapidly determine whether the transposon inserted into the repeat-bearing region of the target plasmid (Figure 1). DNA sequencing using primers from the ends of the Tn7 transposon was then used to determine precisely where the transposon had inserted within the repeat-bearing fragment. To aid in discussing our results, the GAA•TTC repeat was divided into thirds. Repeats 1–36 made up the first third, repeats 37–72 the second, and repeats 73–108 the last (Figure 1). GAA•TTC repeats readily form Y•R•Y DNA triplex structures in supercoiled plasmids [18], [19] particularly when in their native flanking sequence [19]. Given the reported propensity for Tn7 to insert next to Y•R•Y DNA structures [27], [28] we reasoned that encouraging structure formation within the GAA•TTC repeat would lead to an increase in Tn7 insertion. We therefore conducted *in vitro* transposition reactions at pH 7.0 to encourage Y•R•Y DNA structures, as well as at the recommended pH 8.0. Transposition reactions were also conducted at pH 9.0 where Y•R•Y triplex formation is less favored [30], [31]. The expected occurrence of insertion into the repeat was calculated based on the size of the repeat compared to the size of the rest of the plasmid (minus the *bla* gene and the origin of replication). The expected percentage of insertion into the repeat if Tn7 inserts at random was calculated to be about 9%. However, when triplex formation was encouraged at pH 7.0, of ninety-six insertions analyzed, 48% were within the GAA•TTC repeat (Figure 2). At pH 8.0, of 120 insertions analyzed, 39% of insertions occurred within the repeat. At pH 9.0, where the acid stabilized Y•R•Y triplex formation is discouraged, only 14% of 96 insertions occurred within the repeat (Figure 2). As a control *in vitro* transposition system, we chose the EZ::TN system to insert Tn5 into our target plasmids. In stark contrast to Tn7, Tn5 showed no preference for inserting into the GAA•TTC repeat at any pH value tested (Figure 2).

**Figure 3:**
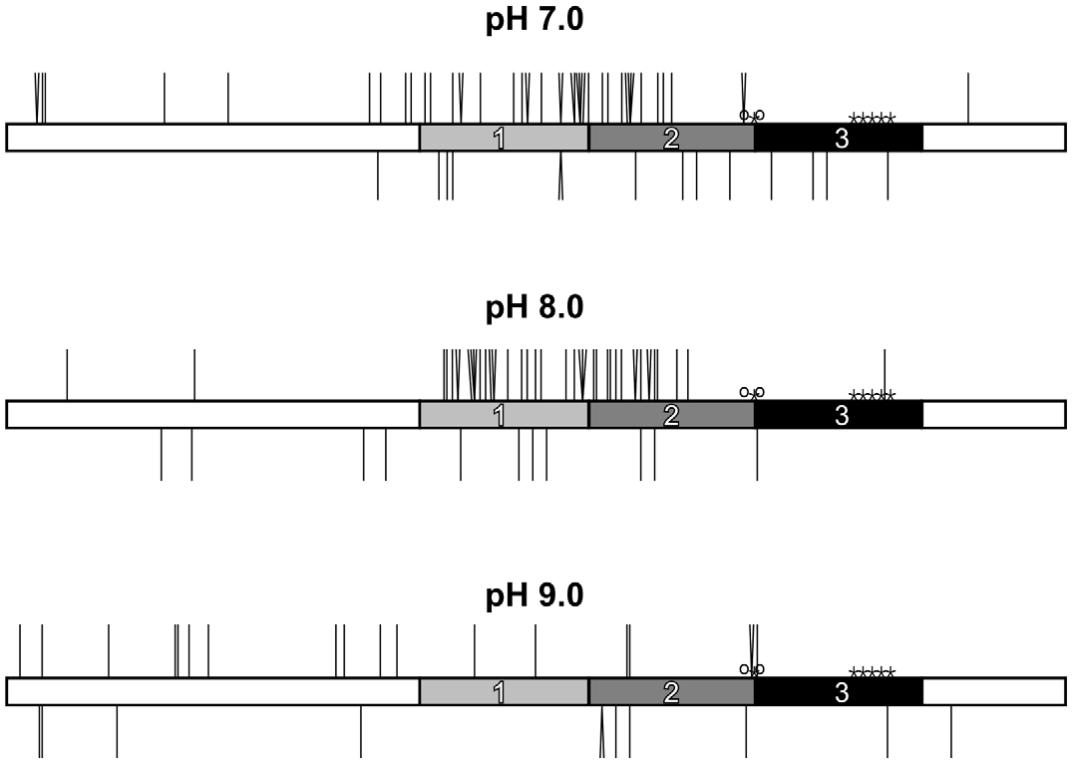
The location and orientation of Tn7 insertion within the GAA•TTC repeat is pH dependent. Depictions of the MluI to XbaI fragment containing the GAA•TTC repeat with vertical lines indicating Tn7 insertion events at pH 7, pH 8 and pH 9 both within and near the repeat. The repeat is divided into thirds, and point deletions and substitutions in the GAA repeat are indicated by open circles and asterisks, respectively. Lines extending above represent insertions events in the (+) orientation, defined as the transposon oriented from left to right. Lines extending below represent insertion events in the (−) orientation with the transposon oriented right to left. Insertions performed at pH 7 and pH 8 show a strong bias for the left to right (+) orientation of the transposon. At pH 7.0 and pH 8.0, Tn7 shows a bias for insertion within the largely uninterrupted regions 1 and 2. In contrast, region 3, an area where the GAA•TTC repeat contains multiple interruptions, is not a favored target for insertion. Location of insertion was determined by DNA sequencing.

Neither transposon inserted into a short normal allele containing only 9 GAA•TTC repeats under any conditions (not shown). Finer analysis of Tn7 insertions within the long GAA•TTC repeat by DNA sequencing showed that an uninterrupted stretch of 18 GAA•TTC repeats located between the interruptions in region 3 of the repeat was also not a frequent target for insertion (Figure 3). The majority of insertions into the repeat at pH 7.0 and pH 8.0 were in the first and second regions. However, when Y•R•Y triplex formation was discouraged by conducting reactions at pH 9.0, there was less bias in the insertion location (Figure 3). Integration into the same base position can indicate a preferred spot, or be the result of analysis of sibling colonies. To limit the number of siblings that were analyzed, we limited the analysis to 24 colonies from individual transposition reactions. In 20 cases, 2 or more transposons were in the same location. In 15 of these cases the transposons were in opposite orientation, or from different transposition reactions, leaving just 5 of the duplicates shown in Figure 3 as potential sibling clones.

### Tn7 shows orientation-specific insertion into GAA•TTC repeats at lower pH

Transposon Tn7 has been shown to exhibit polarity with respect to insertion next to psoralen cross-linked triplex forming oligonucleotide structures [27], [28]. We were also able to discern a polarity with regard to the orientation of Tn7 insertion within the GAA•TTC (Figure 3). When the transposition reaction was performed at pH 7.0 or pH 8.0, more than 75% of total insertions in the first part of the repeat occurred with the transposon in the left to right, or the “plus” orientation relative to the 5′ to 3′ orientation of the GAA strand. For insertions in the second part of the repeat, the majority was also in the “plus” orientation. In contrast, in the third part of the repeat, the few insertions that occurred were mostly in the “minus” orientation. At pH 9.0, there was little bias in orientation for the few insertions that occurred in the repeat (Figure 3).

**Figure 4:**
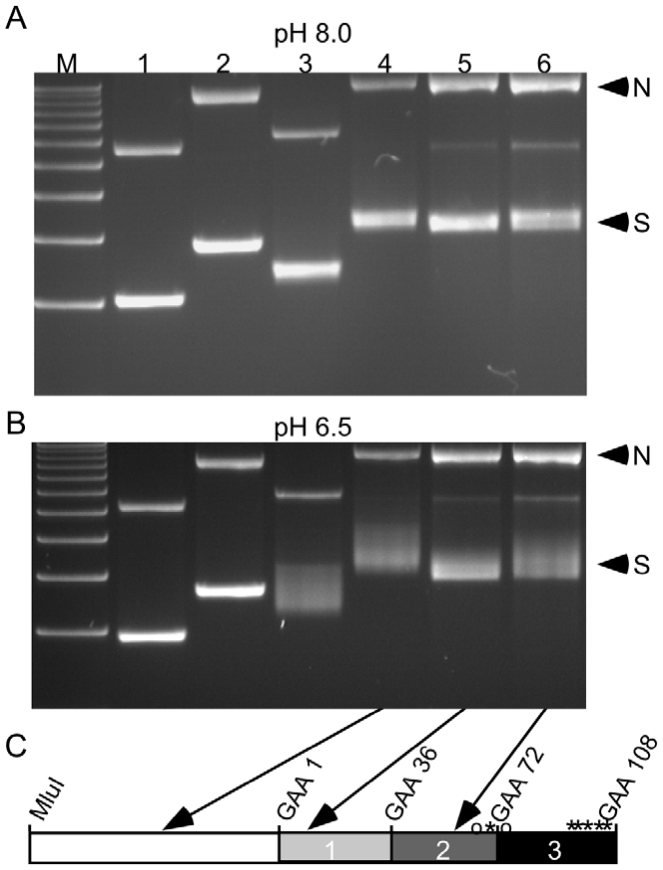
Interruptions in the GAA•TTC repeat alter pH dependent formation of secondary structure. The electrophoretic mobility of nicked (N) and supercoiled (S) plasmids at pH 8.0 and pH 6.5 is shown in A and B, respectively. M = 1KB plus ladder, Lane 1∶9 repeats, no transposon. Lane 2∶9 repeats and Tn5 transposon 5′ of the first repeat. Lane 3∶108 repeats, no transposon. Lane 4∶108 repeats and transposon Tn5 86 bases 5′ of the first repeat. Lane 5∶108 repeats and transposon at repeat 8. Lane 6∶108 repeats and transposon at repeat 57. Arrows indicate the repeat region containing the Tn5 transposon in plasmids run in lanes 4, 5 and 6 on the schematic representation of the repeat target in panel C. The size of the plasmid in lane 1 is 4815 bp; in lane 2, 6328 bp; in lane 3, 5100 bp; in lanes 4–6 6613 bp.

The formation of triplex DNA is able to relax negative supercoils such that covalently closed plasmids can fluctuate between being partially relaxed and supercoiled [18]. We can visualize this as a change in electrophoretic mobility when samples are subject to electrophoresis at a pH compatible with Y•R•Y triplex formation. To determine whether the insertion of a transposon would inhibit triplex formation, we analyzed the electrophoretic mobility of plasmids containing a Tn5 transposon at different areas along the repeat (Figure 4). We used Tn5 for these experiments, because Tn5 inserted at random within the repeat, and we were able to obtain plasmids containing Tn5 at desired locations and orientations along the repeat. At pH 8.0, bands representing the supercoiled forms of samples (S in Figure 4) migrate as relatively tight bands (Figure 4A). However, at pH 6.5, supercoiled samples containing 108 repeats exhibit variable migration indicating partial relaxation due to the formation of DNA secondary structures (Figure 4B, lanes 3–6). When the transposon is inserted adjacent to, but outside the repeat, there is little difference compared to plasmids without transposon insertion (compare lanes 3 and 4). When a transposon is inserted into the repeat, we see a variable decrease in relaxation, depending upon the location of the insertion within the repeat tract. For instance, interrupting the uninterrupted first region of GAA•TTC repeats results in a much closer approximation to normal supercoiled mobility at pH 6.5 (lane 5) compared to insertion near a pre-existing interruption in the second region of the repeat (lane 6).

**Figure 5:**
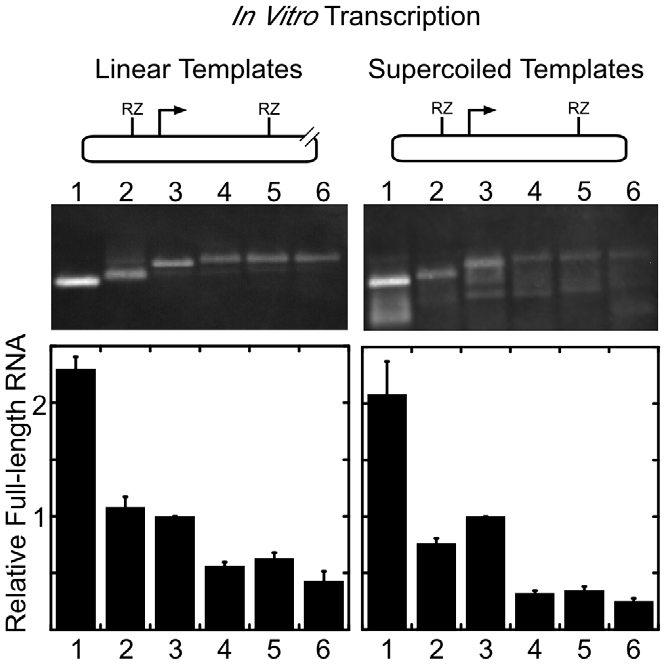
Interrupting the (GAA•TTC)_108_ repeat with a transposon does not improve transcription *in vitro*. The strategy used for *in vitro* transcription of linear and supercoiled templates is summarized in the schematic at the top. Immediately below that is the image of products of *in vitro* transcription of linear and of supercoiled plasmids visualized by hybridization with a biotinylated ODN probe followed by chemiluminescence. The templates are as follows: Lane 1∶9 repeats, no transposon. Lane 2∶108 repeats, no transposon. Lane 3∶9 repeats, Tn5 transposon 5′ to the first GAA. Lane 4∶108 repeats, transposon Tn5 86 bp 5′ of the first GAA. Lane 5∶108 repeats, Tn5 transposon at repeat 8. Lane 6∶108 repeats, Tn5 transposon at repeat 57. The intensity of the bands representing *in vitro* transcription was quantitated with Kodak Molecular Imaging software. Bars represent mean amount of transcription for three independent experiments. A representative blot is shown.

The formation of secondary structures by GAA•TTC may contribute to a block in transcription elongation and reduce the amount of full-length transcript in FRDA [8]–[10]. We have demonstrated in the past that such a block to transcription elongation does occur in simple, *in vitro* T7 transcription systems, and is exacerbated by negative supercoiling in the template [11], [18]. This led us to investigate whether insertion of a transposon would interrupt the repeat to a degree that would allow an increase in transcription. To achieve this, oligodeoxynucleotides were used to insert a T7 promoter next to the repeats in selected clones. Because negative supercoils can help stabilize many non-B DNA structures, we wanted to be able to directly compare the products of transcription from relaxed, linear templates and negatively supercoiled templates. Therefore, a fragment of the plasmids containing the T7 promoter, and the *FXN* repeat region (with or without Tn5 insertions) was moved into a vector containing two self-cleaving ribozymes [32]. The arrangement was such that one ribozyme cut site was located 106 bases upstream of the T7 promoter, and the other 216 bases downstream of the GAA•TTC tract. Thus, self-cleavage of the downstream ribozyme produced identical transcripts from supercoiled templates, or templates made linear by restriction digestion downstream of the ribozyme. We performed *in vitro* transcription on supercoiled and linear templates and compared the transcript yield (Figure 5).

The effect of negative supercoiling on transcript yield was less for this sequence than we had seen in the past with 88 uninterrupted GAA•TTC repeats [11]. However, a small difference is discernible, comparing lanes 1 and 2 for linear or supercoiled templates in Figure 5. Unexpectedly, the presence of the mini Tn5 transposon also attenuated full-length transcription by T7 RNA polymerase, although this was not exacerbated by negative supercoiling (compare lanes 1 and 3 for both linear and supercoiled templates in Figure 5). In contrast to the differential effects seen on Y•R•Y triplex formation in Figure 4, there was little difference seen in transcript yield when a transposon was adjacent to, or within the GAA•TTC repeat (compare lanes 4 with lanes 5 and 6 in Figure 5).

## Discussion

We have shown that structure formation by a Friedreich ataxia patient-derived GAA•TTC repeat tract directs Tn7 transposition to the repeat *in vitro*. The transposon inserts in a region-specific and orientation-specific manner within the repeat in the absence of its usual target specificity proteins TnsD or TnsE, suggesting that structures formed by the repeat somehow replace functions of those specificity factors. In the cell, Tn7 uses two distinct transposition target site selection pathways encoded by five genes (TnsABCDE). TnsA, TnsB and TnsC provide a core recombination machine that mediates the DNA strand breakage and joining reactions [23]. TnsD directs high frequency orientation-specific insertion by TnsABC into a single site in the *E*. coli genome called *attTn7* in a process that involves TnsD distorting the target site [33]. The combination of TnsABC and TnsE promotes insertion into DNA undergoing lagging strand synthesis. In particular, TnsE promotes preferential insertion into transferred conjugal DNA using two critical features: DNA structure and specific binding to the sliding β-clamp [25], [34].

In the absence of TnsD and TnsE, a hyperactive TnsABC* complex directs transposition into many different target sites with low selectivity [26], but has also been shown to recognize and preferentially insert adjacent to psoralen cross-linked triplex forming oligomers [27]. Tn7 insertion is orientation-specific, with the right side of Tn7 towards the cross-linked third strand, and is specific for pyrimidine oligonucleotides bound in a Y•R•Y triplex [28]. In our system, the pH dependence of structure formation by the GAA•TTC repeat that results in Tn7 insertion suggests a Y•R•Y triplex. Long stretches of polypurine-polypyrimidine sequences such as GAA•TTC can adopt an intramolecular Y•R•Y triplex structure sometimes called H-DNA [15], [16], [20]. H-DNA forms when duplex DNA is opened up and the polypyrimidine (Y) strand folds back onto the duplex forming a triple stranded Y•R•Y helix. Two isomers can form: H-y3, and H-y5, depending on whether the part of the Y strand that folds over to become the third strand is from the 3′ or the 5′ end, respectively [20]. We do not know whether H-y3 or H-y5 predominates in the GAA•TTC repeats in our plasmids, although we can predict that structures formed as a consequence of DNA opening in an A and T rich stretch derived from an AluSx element immediately upstream of the GAA•TTC repeat would favor the H-y3 conformation. In addition, we saw that insertion of a Tn5 transposon near that A and T rich stretch had a strong effect on Y•R•Y triplex formation at pH 6.5 in gel mobility assays (see Figure 4, lane 5). Finally, spontaneous opening from within the interrupted, and slightly more GC rich region 3 of the repeat (the 5′ end of the Y strand) would discourage H-y5 structures in that end of the repeat, unlike the uninterrupted GAA•TTC repeats that formed a bi-triplex due to opening at both flanking A•T stretches [19]. The bias in polarity of Tn7 transposition into the repeat suggests that a specific structure may be instigating the insertions. Alternatively, it may indicate that Tn7 tracks on the DNA with a particular orientation. However, the nature of the predominant structures formed in the transposition assay buffer, or the subset of structures responsible for attracting the insertion events must await future studies.

It is noteworthy that Y•R•Y triplex formation by the GAA•TTC repeat is spontaneous and not covalently constrained by psoralen cross-links, yet stable enough to effectively attract Tn7 insertion at pH 7 or even the slightly alkaline pH 8. A hallmark of the Y•R•Y triplex is its pH dependency owing to the need for protonation of the third strand cytosine base in order to form stable Hoogsteen bonds with the central guanine in a C•G•C triplet [15], [16], [20]. However, the GAA•TTC repeat is only one third G•C, so its pH dependency is much less than that of a more G•C rich R•Y sequence. Intramolecular triplexes are additionally stabilized by the energy gained by relaxation of local negative supercoils [16], [35]. Consequently, even in the complete absence of divalent cations during gel electrophoresis we see evidence for substantial Y•R•Y triplex formation at pH 6.5 for the sequence used in this study (see figure 4), and for uninterrupted GAA•TTC sequences we have seen triplex formation at neutral pH [18]. Divalent cations, although not essential for Y•R•Y triplex formation will help stabilize the triplex [16] and the presence of magnesium in the transposition reaction buffer apparently pushed the stability of the triplex up to pH 8.0, judging by the similarity of Tn7 transposition events to those at pH 7.0. However, it is likely that cytosine protonation was not sufficient at pH 9 to support Y•R•Y triplex structures [30], [31] even in the presence of negative supercoiling and a stabilizing divalent cation.

Our data agree with previous work that shows it is likely that GAA•TTC repeats can form the Y•R•Y triplex under physiologically relevant pH and ionic conditions [19]. We speculate that structure formation by the repeat contributes to GAA•TTC repeat instability. Uninterrupted GAA•TTC repeat sequences display high levels of genomic instability, with a tendency towards progressive expansion [6], [7]. Interruptions in the purity of the repeats reduce the expansion rate in human cells. While the work here is a proof of concept that GAA•TTC repeats can be specifically targeted for interruption, we do not suggest that Tn7 could be the basis for any sort of useful therapy. However, our demonstration of a recombinase targeting a spontaneously formed triplex DNA structure under physiologically relevant conditions has wider implications. DNA structure has been described at multiple chromosomal translocation break points [36], particularly in cases where RAG recombinase is involved [37], [38]. It is likely that a number of enzymes involved in DNA transactions are attracted to DNA structure, in addition to Tn7 transposase and RAG recombinase. Future identification of such complexes in human nuclei that target GAA•TTC repeats may provide the key to understanding both why GAA•TTC repeats expand, and how they subsequently repress gene expression.

## Materials and Methods

### PCR amplification of genomic DNA

Primers 517F (5′ GGCTTAAACTTCCCACACGTT 3′ ) and 629R (5′ AGGACCATCATGGCCACACTT 3′) were used in the amplification of human genomic DNA. PCR parameters included 94°C for 2 minutes, 30 cycles of 94°C for 30 seconds, 68°C for 1 minute and 30 seconds plus 10 seconds added every cycle, followed by 72°C for 10 minutes and a 4°C hold. The pH of MOPS (3-[N-morpholino] propanesulfonic acid) is much less temperature dependent than that of Tris and it provides better results for GAA•TTC repeats than standard PCR reactions. Therefore, amplification was done in MOPS + Triton buffer (20 mM MOPS pH 8.2, 2 mM MgSO_4_, 5 mM (NH_4_)_2_SO_4_, 0.1% TritonX-100), 0.25 mM each dNTP, and 1 U Takara Ex enzyme (TaKaRa Bio). PCR products were resolved on a 1% agarose gel containing ethidium bromide in TAE buffer (40 mM Tris-Acetate pH 8.0, 2 mM EDTA). DNA was visualized by UV transillumination.

### Gel Purification

To obtain an expanded *FXN* allele, the PCR product band corresponding to an individual allele was excised from an agarose gel after resolution. The gel slice was centrifuged through spun polyester fiber, phenol chloroform extracted, sodium acetate/ethanol precipitated and resuspended in TE (10 mM Tris pH 8.0, 1 mM EDTA). Secondary amplification, to increase the yield of gel-purified samples, was performed. PCR parameters for secondary amplification were: 94°C for 2 minutes, 15 cycles of 94°C for 1 minute, 64°C for 2 minutes, 72°C for 1 minute 30 seconds, followed by 72°C for 10 minutes and a 4°C hold. Products were resolved and extracted as above.

### Molecular cloning

The plasmid pBad18 [39] was digested with restriction enzymes NheI and XmaI. Purified secondary amplifications of genomic samples were digested with restriction enzymes AvrII and XmaI. The double digested vector and PCR products were gel purified. A 1∶1 vector to insert molar ratio was ligated using T4 ligase (Invitrogen) and transformed into Xl-1Blue MRF' (Stratagene). Transformants were selected using ampicillin (100 µg/ml). Plasmids were isolated using alkaline lysis as described [40] unless otherwise indicated. Ligations resulted in a pBad18 plasmid containing either 9 or 108 GAA•TTC repeats and 5′ and 3′ flanking regions contain 266 and 168 bases, respectively of *FXN* sequence. The size of the plasmids was 4815 base pairs and 5100 base pairs, respectively. The sequence of the cloned, expanded allele is available online (GenBank accession number GU722204).

### Transposon Insertion

The GPS-1 *in vitro* transposition system (New England Biolabs) was used as directed by the manufacturer to insert transposon Tn7 into plasmids containing 9 or 108 GAA•TTC repeats. Briefly, pGPS2.1 donor DNA was incubated with target DNA (0.08 µg) in the presence of TnsABC* transposase in GPS buffer (25 mM Tris-HCl pH 8.0, 2 mM DTT and 2 mM ATP) for 10 minutes at 37°C. The pH of the GPS buffer was adjusted to pH 7.0 and pH 9.0 for some experiments. Start solution (15 mM magnesium acetate, final concentration) is added and incubated at 37°C for 1 hour. After heat inactivation at 75°C for 10 minutes, the reaction was transformed into Xl-1Blue MRF' cells. Plasmids were selected using ampicillin (100 µg/ml) with chloramphenicol (15 µg/ml). The transposon donor plasmids had an R6K-γ origin of replication and do not replicate in Xl-1 Blue. 24 colonies were analyzed from each transposition experiment. Transposon location was first mapped by MluI/XmaI digestion. The location of transposition events in the smaller MluI/XmaI fragment (in or near the repeat) was determined by DNA sequencing using primer N (5′ ACTTTATTGTCATAGTTTAGATCTATTTTG 3′) and primer S (5′ ATAATCCTTAAAAACTCCATTTCCACCCCT 3′) of the GPS system.

The EZ::TN<Kan-2> *in vitro* insertion system (Epicentre) was used as directed by the manufacturer to insert a Tn5 transposon into target plasmids containing 9 or 108 GAA•TTC repeats. Briefly, target DNA was incubated with EZ::TN™<KAN-2> transposon in the presence of EZ::TN transposase in reaction buffer (50 mM Tris-Acetate pH 8.0, 150 mM potassium-acetate, 10 mM magnesium acetate, and 4 mM spermidine) for 2 hours at 37°C. The pH of the EZ-TN reaction buffer was adjusted to pH 7.0 or pH 9.0 for some experiments. The reaction was stopped by adding of stop solution (0.1% SDS, final concentration), mixed and heated for 10 minutes at 70°C. The mixture was then transformed into E. coli Xl-1Blue MRF' competent cells and selected using ampicillin (100 µg/ml) with kanamycin (50 µg/ml). The transposon donor plasmids had an R6K-γ origin of replication and do not replicate in Xl-1 Blue. 24 colonies were analyzed from each transposition experiment. Transposon location was first mapped by MluI/XmaI digestion. The location of transposition events in the smaller MluI/XmaI fragment (in or near the repeat) was determined by DNA sequencing using primers Kan2-FP-1 (5′ ACCTACAACAAAGCTCTCATCAACC 3′) and Kan2-RP-1 (5′ GCAATGTAACATCAGAGATTTTGAG 3′) of the EZ-TN system.

### Addition of T7 promoter to selected plasmids

When annealed, oligonucleotides T7a (5′ CCGCTAATTAATACGACTCACTATAGGGA 3′) and T7b (5′ CGCGTCCCTATAGTGAGTCGTATTAATTA 3′) leave sticky ends corresponding to MluI and AgeI restriction sites. The oligonucleotides (10 µM, final concentration) were mixed in 50 mM NaCl in TE at 75°C. The temperature was gradually cooled allowing oligonucleotides to anneal. A 1∶10,000 dilution of the annealed linker was ligated to AgeI/MluI digested plasmids.

### Cloning between self-cleaving ribozymes

Plasmids containing the inserted T7 promoter were digested with XmaI and AgeI, to release the repeats (some interrupted with transposon Tn5) linked to the T7 promoter. A plasmid containing two self-cleaving ribozymes in tandem, TAN1 [32] was linearized by restriction digest with XmaI, which cuts between the two ribozymes. Phosphates were removed from the cut ends of the TAN1 plasmid with bacterial alkaline phosphatase (BAP). The repeat inserts were then ligated to XmaI cut TAN1. Construct integrity was verified by sequencing with primers 2515a (5′ CGTCGCCAGTCAAGTAACAA 3′) and 3232b (5′ CCAAAAGACGGCAATATGGT 3′) that prime from within the TAN1 vector. Aliquots of these templates described in the figure legends were then used for *in vitro* transcription. The size in base pairs (bp) of these transcription vectors was as follows: TAN1-9GAA, 9108 bp; TAN1-108GAA, 9403 bp; TAN1-9GAA-Tn5, 10621 bp; TAN1-108GAA-Tn5, 10916 bp.

### Gel mobility assay

Samples were separated by electrophoresis on a 1% agarose gel in TAE buffer (40 mM Tris-Acetate pH 8.0, 2 mM EDTA) for 5 hours at a constant voltage of 60v. Electrophoresis was conducted at pH 6.5, and pH 8.0 in a buffer puffer (Owl Separation Systems) recirculating electrophoresis box in order to maintain a uniform pH. Gels were stained with ethidium bromide for visualization after electrophoresis.

### *In vitro* transcription

Concentration matched templates, either supercoiled or linearized by restriction digestion with AvrII (which cuts 100 bases downstream of the second ribozyme cleavage site in TAN1) were prepared. Aliquots were added to T7 transcription buffer (50 mM HEPES pH 8.0, 100 mM NaCl, 20 mM MgCl2, 10 mM DTT and 0.5 mM each NTP), and T7 RNA polymerase containing RNase inhibitor (Ambion) was added to start the reactions. Samples were transcribed for 30 minutes at 37°C. The reaction was stopped with the addition of loading buffer containing 5 mM EDTA. Electrophoresis was done on a 1% agarose gel containing ethidium bromide in TAE buffer pH 8.0. Samples were then transferred to a nylon membrane overnight.

### Northern blot

Following transfer, the membrane was UV cross-linked for one minute. The blot was washed 2X 5 minutes in 2XSSC, 0.1% SDS followed by pre-hybridization in 7% SDS, 0.5M NaPO_4_ pH 7.5 and 1 mM EDTA for 30 minutes at 55°C. A biotinylated DNA probe, biomlu (5′ TTTCTGCCGTGATTATAGACACTTTTGTTACGCGT 3′), designed to bind 296 to 260 bases upstream of the first repeat was denatured at 100°C for 10 minutes and then added to the blot in hybridization buffer at a final concentration of 0.03 µM. The probe was allowed to bind for 30 minutes at 55°C, followed by a decrease in temperature to 45°C for 30 minutes, and 37°C for 30 minutes. The membrane was then washed 2X 5 minutes in 2X SSC, 0.1% SDS and then washed 5 minutes in TBST (10 mM Tris pH 8.0, 150 mM NaCl and 0.05% Tween 20). The membrane was blocked overnight in TBST with 0.5% Hammerstein grade casein. Horseradish peroxidase-avidin (0.125 µg/µl final concentration) was then added for 1 hour, followed by washing 3X 5 minutes in TBST. Chemiluminescence using ECL Advance (Amersham) was then used to detect probe binding. Images of the blots were obtained using a Kodak Gel Logic 440 imaging system. Analysis and quantitation was performed with Kodak Molecular Imaging software.
